# Attenuation of Notch and Hedgehog Signaling Is Required for Fate Specification in the Spinal Cord

**DOI:** 10.1371/journal.pgen.1002762

**Published:** 2012-06-07

**Authors:** Peng Huang, Fengzhu Xiong, Sean G. Megason, Alexander F. Schier

**Affiliations:** 1 Department of Molecular and Cellular Biology, Center for Brain Science, Harvard Stem Cell Institute, Broad Institute, Center for Systems Biology, Harvard University, Cambridge, Massachusetts, United States of America; 2 Department of Systems Biology, Harvard Medical School, Boston, Massachusetts, United States of America; University of Pennsylvania School of Medicine, United States of America

## Abstract

During the development of the spinal cord, proliferative neural progenitors differentiate into postmitotic neurons with distinct fates. How cells switch from progenitor states to differentiated fates is poorly understood. To address this question, we studied the differentiation of progenitors in the zebrafish spinal cord, focusing on the differentiation of Kolmer-Agduhr″ (KA″) interneurons from lateral floor plate (LFP) progenitors. In vivo cell tracking demonstrates that KA″ cells are generated from LFP progenitors by both symmetric and asymmetric cell divisions. A photoconvertible reporter of signaling history (PHRESH) reveals distinct temporal profiles of Hh response: LFP progenitors continuously respond to Hh, while KA″ cells lose Hh response upon differentiation. Hh signaling is required in LFP progenitors for KA″ fate specification, but prolonged Hh signaling interferes with KA″ differentiation. Notch signaling acts permissively to maintain LFP progenitor cells: activation of Notch signaling prevents differentiation, whereas inhibition of Notch signaling results in differentiation of ectopic KA″ cells. These results indicate that neural progenitors depend on Notch signaling to maintain Hh responsiveness and rely on Hh signaling to induce fate identity, whereas proper differentiation depends on the attenuation of both Notch and Hh signaling.

## Author Summary

During tissue formation, progenitor cells generate both differentiated cells and progenitor cells. It is poorly understood how this balance between self-renewal and differentiation generates the correct number of different cell types. Here, we use zebrafish spinal cord development as a model system to investigate how neural progenitor cells switch from progenitor states to differentiated fates. Combining genetic manipulation and a novel method to study cell signaling in live embryos, our data show that this process requires the dynamic regulation of two signaling pathways: the Notch signaling pathway and the Hedgehog (Hh) signaling pathway. In neural progenitors, Notch signaling maintains the competence of neural progenitors to respond to Hh signaling. In parallel, Hedgehog signaling functions to induce cell fate identity. As cells switch from progenitor states to differentiated states, both Notch and Hh signaling become attenuated. Thus, the dynamic deployment of Notch and Hh signaling controls the renewal and differentiation of progenitor cells.

## Introduction

During spinal cord development, proliferative neural progenitors arrayed along the dorsal-ventral axis differentiate into postmitotic neurons with distinct functions and morphologies [1]–[3]. Each dorsal-ventral domain consists of both neural progenitors and differentiated neurons. For example, the mouse V3 domain immediately dorsal to the floor plate contains medially located V3 progenitor cells and laterally located differentiated V3 interneurons [4]. Analogously, the lateral floor plate (LFP) in zebrafish contains two one-cell-wide domains flanking the centrally located medial floor plate [5]–[8]. Within each LFP domain, LFP progenitors, early-born Kolmer-Agduhr″ (KA″) interneurons, and late-born V3 interneurons are distributed in a discontinuous pattern along the anterior-posterior axis [5], [6].

Hedgehog (Hh) and Notch signaling play important roles in spinal cord patterning [3]. Sonic hedgehog (Shh) is the key inductive signal that patterns the ventral spinal cord [1]. It functions by binding to its receptor Patched (Ptc) and relieves the inhibition of Smoothened (Smo). Activation of Smo initiates a downstream signaling cascade that leads to the activation of the Gli family of transcription factors. During spinal cord development, Shh is secreted by the notochord and floor plate. The gradient of Hh signaling activity regulates the expression of a number of transcription factors in neural progenitors. The combinatorial expression of these transcription factors defines distinct progenitor domains along the dorsal-ventral axis that give rise to V0, V1, V2 interneurons, motor neurons (MN), V3 interneurons, and the floor plate [1]. In addition to Shh concentration, the duration of Hh signaling also contributes to the patterning of the ventral spinal cord [9], [10]. For example, induction of *nkx2.2*, a marker for the ventral V3 precursor domain, requires a higher concentration and a longer duration of Shh signaling compared to *olig2*, a marker for the more dorsal MN precursor domain [9]. Thus, the level and duration of Hh signaling assign distinct fates along the dorsal-ventral axis of the spinal cord.

Notch signaling has also been implicated in neural development [11], [12]. Activation of Notch signaling results from interaction of Notch receptors with their ligands Delta and Jagged [13]. Upon receptor activation, the Notch intracellular domain (NICD) is cleaved and translocates to the nucleus to associate with the DNA binding protein CBF1 (RBP-J/CSL) to activate the transcription of target genes. Components of the Notch signaling pathway are expressed in distinct domains in the spinal cord [14]–[21]. One major function of Notch signaling is to maintain neural progenitor state by preventing the expression of proneural genes [11], [12]. For example, conditional knockout of Notch1 in neural progenitor cells results in the progressive loss of all subtypes of progenitor cells in the ventral spinal cord [22]. Similarly, in zebrafish *deltaA* mutants, neural precursors differentiate into early-born primary motor neurons at the expense of late-born neurons [17]. Conversely, constitutive activation of Notch signaling prevents neuronal differentiation [23]. Thus, Notch signaling maintains progenitors in the spinal cord.

Despite the well-established roles of Hh signaling in fate specification and of Notch signaling in progenitor maintenance, it is unclear how these signaling pathways interact to orchestrate neuronal patterning. Several Notch ligands show domain-specific expression that is controlled by transcription factors downstream of Hh signaling [15], [16], [21]. For instance, Nkx6.1 and Dbx1 function together to establish the expression of Jagged1 in the V1 precursor domain and Delta1 in the motor neuron, V2, and V0 precursor domains [15], [16]. Loss of Delta1 or Jagged1 leads to a domain-specific increase in neuronal differentiation, but does not affect the establishment of progenitor domains [15], [16]. These results suggest that Hh signaling acts upstream of Notch signaling in patterning of the ventral spinal cord. In support of this model, activation of Hh signaling in neural progenitors of the neocortex by *Ptc1* deletion induces the expression of Notch target genes and promotes proliferative divisions. This phenotype can be suppressed by concomitant attenuation of Notch signaling [24]. By contrast, Shh induces the expression of ventral neuronal markers in neuralized embryoid bodies (EBs) regardless of Notch pathway activity [25]. This result suggests that Notch and Hh signaling function in parallel during neuronal differentiation.

Both Hh and Notch signaling have been implicated in the specification of KA″ interneurons in the lateral floor plate domain [6], [18]. Shh is expressed in the medial floor plate, and induces the expression of homeodomain transcription factors, including *nkx2.2a*, *nkx2.2b*, and *nkx2.9*, in the LFP domain [5], [6], [8], [26]. Nkx2.2a, Nkx2.2b, and Nkx2.9 function redundantly to activate the expression of a cascade of transcription factors, including Gata2 and Tal2, to specify KA″ identity [5]. The intermixing of LFP progenitors and KA″ interneurons has led to the suggestion that their differential specification might result from different sensitivity to Shh [6]. In particular, LFP cells require high levels of Hh signaling, while KA″ cells require lower levels [6].

In addition to Hh signaling, Notch signaling also plays a role in KA″ specification [6], [18]. Loss of Notch signaling in the mutant *mindbomb* (*mib*), which encodes a ring type ubiquitin ligase required for Delta activity, results in loss of both LFP and KA″ cells [6]. By contrast, morpholino knockdown of *jagged2*, which is expressed in the dorsal motor neuron domain, induces ectopic KA″ cells [18]. It has been suggested that Jagged2 interacts ventrally with LFP progenitors to prevent the differentiation from LFP progenitors to KA″ interneurons [18]. However, it remains unclear how Notch and Hh signaling interact in KA″ specification.

Here we determine the role of Hh and Notch signaling in the specification of KA″ interneurons in zebrafish. Using in vivo time-lapse imaging, we demonstrate that KA″ cells can be generated from LFP progenitors in both symmetric and asymmetric cell divisions. To map the temporal profile of Hh response at single-cell resolution, we developed a novel technique (PHRESH) using a photoconvertible Hh signaling reporter, *Ptc1-Kaede*. Surprisingly, despite sharing common progenitors, KA″ cells terminate Hh response upon differentiation while LFP cells remain Hh responsive. By manipulating Hh and Notch activity, we show that neural progenitor cells require Notch signaling to maintain Hh responsiveness and rely on Hh signaling to induce progenitor identity, whereas the downregulation of both Notch and Hh signaling is required for proper differentiation.

## Results

### Lineage analysis of KA″ cells

**Figure 1:**
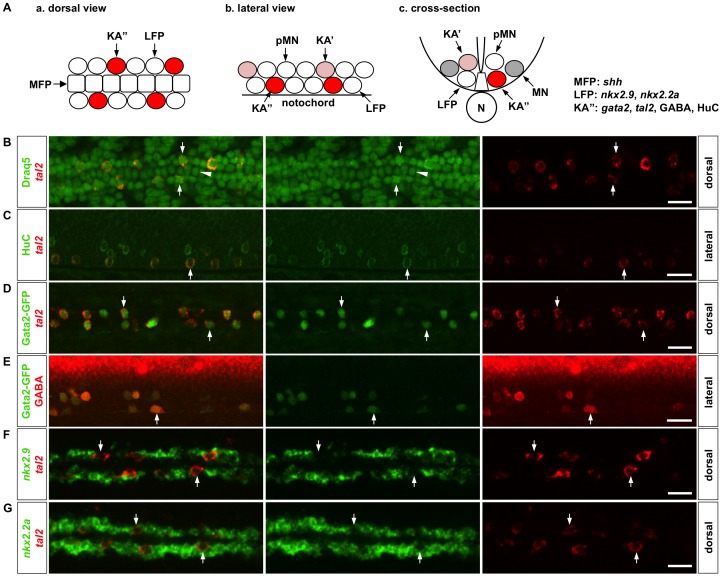
Marker analysis of the LFP domain. (A) Schematic drawings of the zebrafish ventral spinal cord at 24 hpf in a dorsal view (a), lateral view (b), and cross-section (c). The lateral floor plate domain, consisting of LFP cells and KA″ interneurons, flanks the medial floor plate (MFP). The motor neuron domain locates dorsal to the LFP domain, which contains motor neuron progenitors (pMN), motor neurons (MN), and Kolmer-Agduhr′ interneurons (KA′). KA interneurons, including KA′ and KA″ cells, represent a unique class of GABAergic interneurons that are in contact with the cerebrospinal fluid and are implicated in regulating the swimming behavior in zebrafish [35], [47], [48]. Marker expression in MFP, LFP and KA″ cells are indicated on the right. N: notochord. (B) KA″ cells, labeled by *tal2* expression (arrows, red), are discontinuously distributed flanking the medial floor plate (arrowhead) along the anterior-posterior axis. Cell nuclei were labeled by Draq5 staining (green). (C) All *tal2*-positive cells (arrows, red) in the LFP domain also express pan-neuronal marker HuC (green). (D–E) All GFP-expressing cells in the LFP region (arrows, green) in *Gata2-GFP* fish also co-express *tal2* (D, red) and GABA (E, red). (F–G) Wild type embryos were co-labeled with *nkx2.9* (F, green) or *nkx2.2a* (G, green) together with *tal2* (red). *tal2*-expressing KA″ cells (arrows) show lower expression of *nkx2.9* and *nkx2.2a* compared to their neighboring cells. Embryos shown are at 21 hpf (B), 30 hpf (C), 25 hpf (D), 27 hpf (E), and 18 hpf (F, G). B, D, F, and G show dorsal views, and C and E represent lateral views. Scale bars: 20 µm.

We used the lateral floor plate as a model system to study how differentiated cells are generated from progenitor cells. First, we confirmed the heterogeneous nature of the LFP domain using in situ hybridization and immunohistochemistry (Figure 1). By 1 day post fertilization (dpf), two distinct cell types can be distinguished by specific marker expression (Figure 1A). KA″ cells are marked by the expression of *gata2*, *tal2*, the neurotransmitter GABA, and HuC, a marker of differentiated neurons (Figure 1B–1E). They are discontinuously distributed along the anterior-posterior axis. By contrast, *nkx2.9* and *nkx2.2a* are expressed in a pattern complementary to *tal2* expression, with little expression in *tal2*-positive KA″ cells (Figure 1F and 1G). Since both *nkx2.9* and *nkx2.2a* are required for the induction of KA″ cells [5], these results suggest that *nkx2.9* and *nkx2.2a* label undifferentiated LFP progenitor cells and are downregulated in differentiated KA″ cells marked by HuC and GABA.

**Figure 2:**
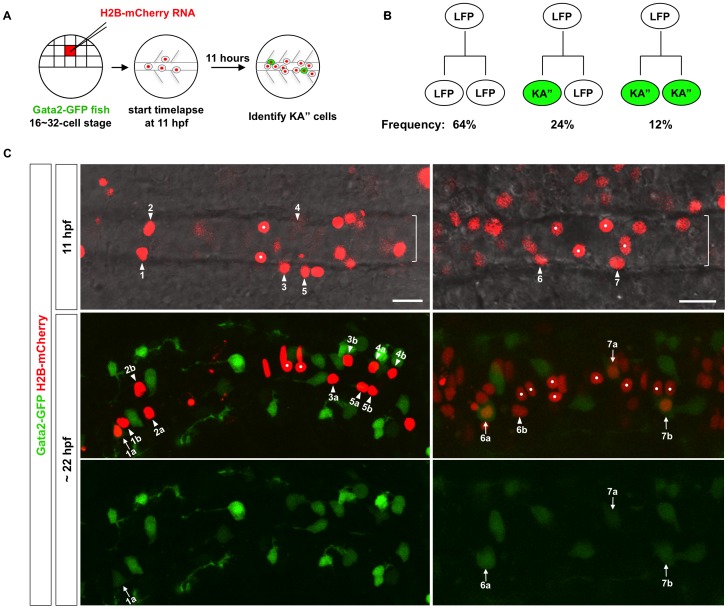
Lineage analysis of the LFP domain. (A) Schematic drawings of scatter labeling and time-lapse imaging. *Gata2-GFP* fish were injected with *H2B-mCherry* mRNA (red) into a single blastomere at 16- to 32-cell stage. Scatter labeled embryos with nuclear mCherry expression (red) were imaged in the dorsal view starting at 3-somite stage (11 hpf) for about 11 hours. At the end of the time-lapse, an image with both the green and red channels was acquired to identify *Gata2-GFP*-expressing KA″ cells (green). (B) Observed division patterns in the LFP domain. Of a total of 25 cell divisions tracked, 16 were LFP/LFP divisions, 6 were KA″/LFP divisions, and 3 were KA″/KA″ divisions. KA″ cells (green) were identified base on the expression of *Gata2-GFP* reporter. (C) *Gata2-GFP* fish (green) was scatter labeled by *H2B-mCherry* (red) and imaged from 11 hpf for about 11 hours. Two examples are shown. Top panel: merged images with both the red channel and the bright field of a single optical slice at the start of the movie at 11 hpf. The underlying notochord (brackets) is visible but out of focus. Middle and bottom panels: the merged image with both green and red channels and the green channel alone of a confocal projection at the end of the movie around 22 hpf. KA″ cells (arrows) can be distinguished from LFP cells (arrowheads) based on the expression of *Gata2-GFP* (green) at 22 hpf. Medial floor plate cells are indicated by white dots. Lineage related cells confirmed by cell tracking are indicated (also see Figure S1 and Video S1). For example, cell 1 generates a KA″ cell (cell 1a) and an LFP cell (cell 1b). Of the 7 examples shown here, cells 1 and 6 undergo KA″/LFP divisions, cell 7 undergoes KA″/KA″ division, and cells 2–5 divide symmetrically giving rise to two LFP cells (LFP/LFP divisions). Note that cells 4 and 6 are more dorsally located and therefore not in focus in images at 11 hpf (top panels). Scale bars: 20 µm.

The discontinuous distribution of KA″ cells in the LFP domain suggests two models for KA″ formation. First, KA″ cells and LFP cells might be generated separately from distinct pools of progenitor cells, and subsequent cell intercalation results in the characteristic “salt-and-pepper” pattern. Alternatively, a LFP progenitor might give rise to both KA″ cells and LFP cells. To test these models, we carried out confocal time-lapse microscopy to assess cell lineages within the LFP domain (Figure 2, Figure S1, and Video S1). Cells were tracked at high temporal resolution using the nuclear marker, *H2B-mCherry*. A *Gata2-GFP* transgenic reporter [27] was used to identify KA″ cells at the end of the time-lapse movie (Figure 1 and Figure S2). LFP cells were identified based on their lateral juxtaposition to the medial floor plate and their anterior or posterior juxtaposition to KA″ cells (Figure 1 and Figure S2). To achieve reliable cell tracing, we generated scatter labeled embryos by injecting *H2B-mCherry* RNA into a single blastomere at the 16-cell to 32-cell stage (Figure 2A). This method allowed us to unambiguously identify cell divisions, trace cell movements and determine the fate of daughter cells (Video S1). By retrospective cell tracing, we identified the distribution of LFP progenitor cells at the early somite stage (11 hpf). At this stage LFP progenitors were positioned both medial-laterally and dorsal-ventrally (Figure 2C, top panels). Progeny of LFP progenitor cells eventually converged to align as two rows of one-cell-wide domains flanking medial floor plate cells (Figure 2C, middle and bottom panels). At 22 hpf, the positions of the two daughter cells generated by a LFP progenitor cell division were quite variable. Some siblings remained immediate neighbors, whereas others were separated by a few cells or even by the midline (Figure 2C, middle and bottom panels). Of 25 divisions imaged within the LFP domain, 64% generated two *Gata2-GFP^−^* LFP cells (LFP/LFP), 24% gave rise to one KA″ cell and one LFP cell (KA″/LFP), and 12% generated two *Gata2-GFP^+^* KA″ cells (KA″/KA″) (Figure 2B). These results reveal that KA″ cells can be generated by both symmetric and asymmetric cell divisions from LFP progenitors.

### Attenuated Hh response in KA″ cells

**Figure 3:**
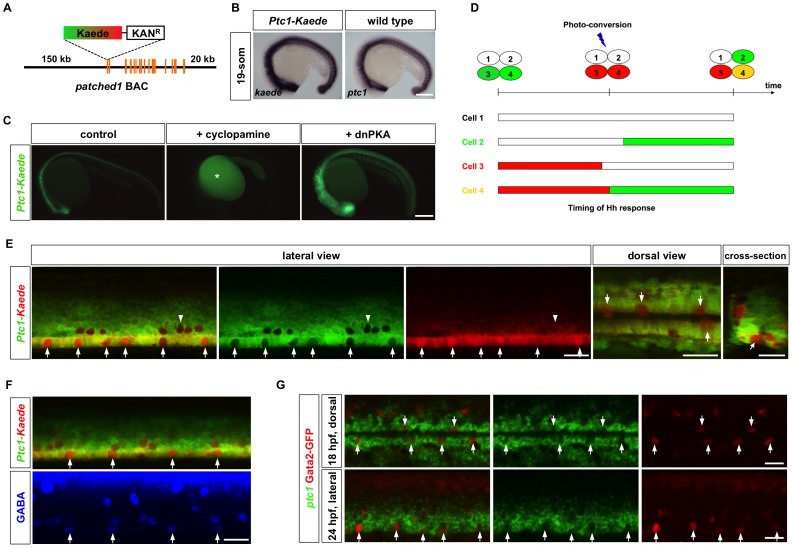
Temporal profiles of Hh response visualized by a *Ptc1-Kaede* reporter. (A) Schematic drawing of the *Ptc1-Kaede* BAC reporter. A cassette containing Kaede and Kanamycin resistant gene was recombined to replace the first exon of *ptc1*. (B) *Ptc1-Kaede* fish showed *kaede* expression in a pattern similar to the expression of *ptc1* in wild type embryos at 19-som stage. (C) Inhibition of Hh signaling using cyclopamine blocked *Ptc1-Kaede* expression, while overexpression of dnPKA mRNA induced ectopic expression of the transgene. Fluorescent signal in cyclopamine-treated fish is due to auto-fluorescence of the yolk (asterisk). (D) Schematic drawings of PHRESH analysis. Photoconversion of the *Ptc1-Kaede* reporter can be used to determine the timing of Hh response (see text for details). (E) *Ptc1-Kaede* fish were photoconverted at 25 hpf, and imaged at 36 hpf. Single optical sections of a lateral view, dorsal view, and cross-section are shown. Arrows indicate *Ptc1-Kaede^red^* cells in the lateral floor domain. Note that dorsally located KA′ cells also only express *Ptc1-Kaede^red^* (arrowheads). (F) *Ptc1-Kaede* fish were photoconverted at 24 hpf, and stained with the GABA antibody (blue) at 35 hpf. Arrows indicate GABA-positive KA″ cells. (G) *Gata2-GFP* fish were co-labeled with *ptc1* (green), and the GFP antibody (red). Images shown are the dorsal view of an 18 hpf embryo (top) and the lateral view of a 24 hpf embryo (bottom). KA″ cells are indicated by arrows. Scale bars: 200 µm in B–C and 20 µm in E–G.

The discontinuous organization of KA″ cells along the LFP domain raises the question how cells with common progenitors acquire different fates. Hh signaling is important in patterning the ventral spinal cord in zebrafish [26], [28]. Since cells in the LFP domain appear to be exposed to similar levels of Shh, it is possible that different duration of Hh signaling in sibling cells results in different fates. To visualize Hh signaling dynamics in vivo, we generated a reporter line for *ptc1*, a direct target of Hh signaling [29]. The cDNA encoding the photoconvertible fluorescent protein Kaede was engineered into a BAC (bacteria artificial chromosome) containing the *ptc1* genomic region, including 150 kb upstream and 20 kb downstream regulatory sequences (Figure 3A). The *Ptc1-Kaede* reporter faithfully recapitulated endogenous *ptc1* expression and responded to Hh signaling (Figure 3B and 3C). Inhibition of Hh signaling using cyclopamine, a potent antagonist of Smo [30], inhibited *Ptc1-Kaede* expression, while overexpression of dnPKA mRNA induced substantial expansion of the expression domain (Figure 3C). These results indicate that the *Ptc1-Kaede* reporter is a sensitive readout for Hh response in vivo.

Taking advantage of the photoconvertible property of the *Ptc1-Kaede* reporter, we developed a novel method (PHRESH, photoconvertible reporter of signaling history) to delineate the duration of Hh response in the spinal cord (Figure 3D). All Hh-responding cells labeled by the green-fluorescent Kaede protein can be photo-converted to red-fluorescent Kaede at a specific time of development. If cells have terminated Hh signaling before the time of photoconversion, they will remain red due to perdurance of the converted red-fluorescent Kaede protein (*Ptc1-Kaede^red^*). By contrast, if cells continue to respond to Hh signaling, they will express *de novo* synthesized unconverted green-fluorescent Kaede and therefore appear yellow (*Ptc1-Kaede^red+green^*). Finally, cells that turn on Hh signaling after photoconversion will appear green (*Ptc1-Kaede^green^*). Therefore, compared to conventional approaches such as GFP reporters, immunohistochemistry or RNA in situ hybridization, the Kaede reporter allows the mapping of Hh signaling history at single cell resolution in live embryos.

To determine the dynamics of Hh response in the LFP region, we performed PHRESH analysis by photoconverting the spinal cord of *Ptc1-Kaede* fish at 24 hpf. At 12 hours-post-conversion (hpc), the LFP region displayed a striking discontinuous pattern of Hh response (Figure 3E and Video S2): some cells had only red fluorescence, whereas their immediate neighbors had both red and green fluorescence. The distribution of *Ptc1-Kaede^red^* cells along the LFP domain was reminiscent of the organization of KA″ cells. To determine their identity, we stained photoconverted fish with the GABA antibody, which specifically labels KA″ cells in the LFP domain. Remarkably, all *Ptc1-Kaede^red^* cells in the LFP domain of photoconverted fish were positive for GABA, and vice versa, suggesting that all KA″ cells terminate their Hh response well before the neighboring LFP cells (Figure 3F). To further confirm this finding, we examined endogenous *ptc1* expression using fluorescent in situ hybridization. Consistent with PHRESH analysis, we found that *Gata2-GFP*-positive KA″ cells had clearly lower levels of *ptc1* transcript compared to the neighboring LFP cells (Figure 3G). These results reveal distinct temporal profiles of Hh response within the LFP domain: LFP cells continuously respond to Hh while KA″ cells lose Hh response. Considering that some LFP progenitors give rise to both KA″ and LFP cells, these results suggest that sibling cells can have distinct Hh responses after division.

### KA″ specification depends on Hh signaling

**Figure 4:**
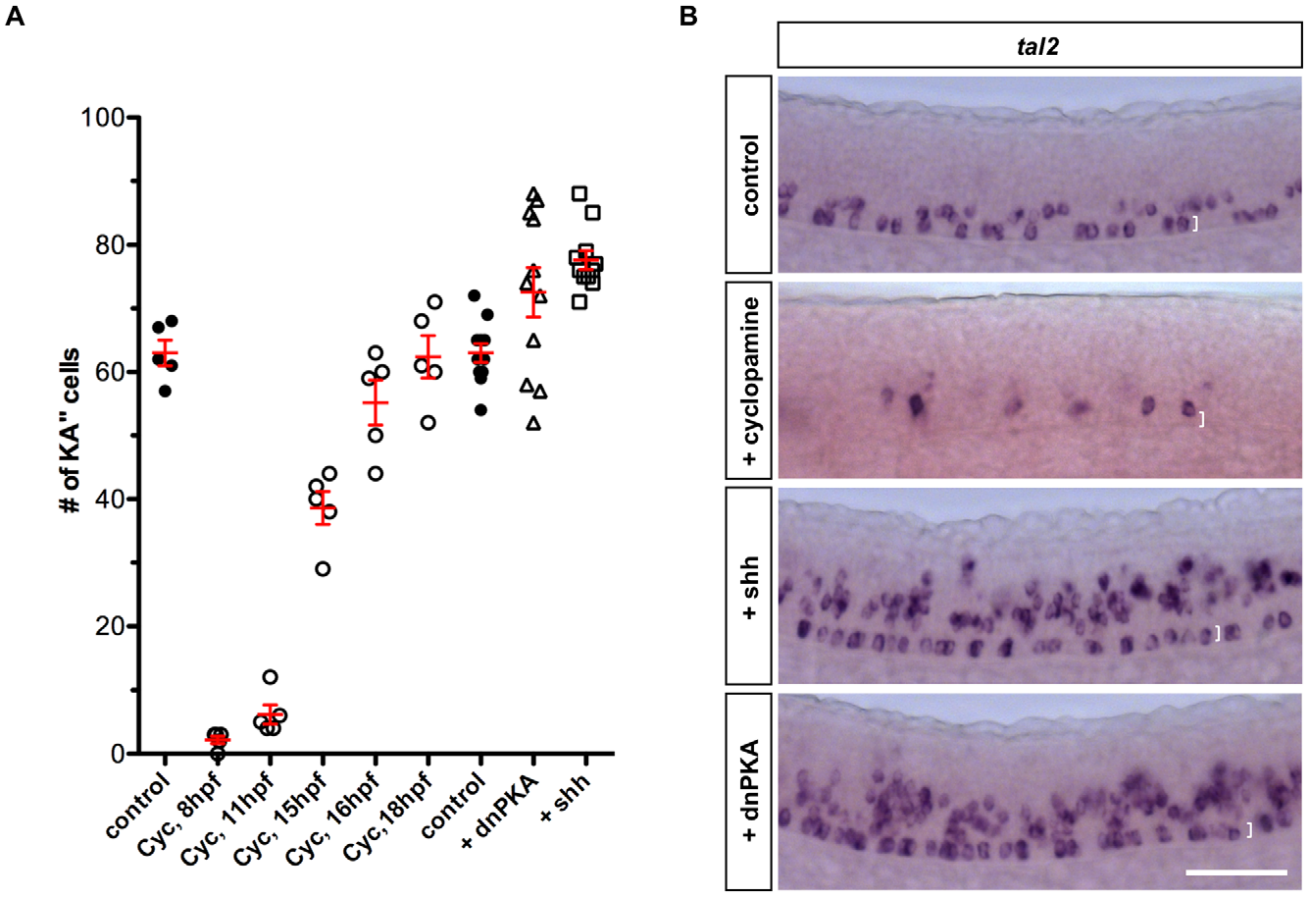
KA″ specification depends on Hh signaling. (A) Quantification of the total number of KA″ cells at 24 hpf under different conditions. Cyclopamine (Cyc) was treated from indicated stage until 24 hpf. Embryos injected with dnPKA or Shh mRNA showed increase in total number of KA″ cells compared to uninjected control embryos. (B) Cyclopamine-treated embryos (from 8 hpf to 24 hpf) abolished *tal2* expression in the LFP domain, while overexpression of dnPKA and Shh induced ectopic *tal2^+^* cells. Note that the few *tal2^+^* cells in cyclopamine-treated embryos are located dorsal to the LFP domain. Images shown are lateral views of embryos at 24 hpf. The dorsal-ventral extent of the LFP domain is indicated by brackets. Scale bar: 50 µm.

PHRESH analysis suggested that Hh signaling is active in progenitors of KA″ cells but absent in differentiated KA″ interneurons. To determine when Hh signaling is required for the generation of KA″ cells, we inhibited Hh signaling using cyclopamine at different stages of development. Inhibition of Hh signaling from 8 hpf to 25 hpf completely abolished *tal2* expression within the LFP domain at 25 hpf (Figure 4A and 4B), suggesting that Hh signaling is required for KA″ induction. By contrast, more than half of KA″ cells were generated when Hh signaling was blocked from mid-somitogenesis (15 hpf) until 25 hpf, while a normal number of KA″ cells formed upon cyclopamine treatment after 18 hpf (Figure 4A). These results suggest that Hh signaling is required in the LFP progenitors before 18 hpf to specify the KA″ fate and that differentiated KA″ cells no longer depend on active Hh signaling. Notably, we did not observe an increase in the number of KA″ cells upon cyclopamine treatment (Figure 4A), indicating that early termination of Hh signaling in LFP cells is not sufficient to transform them into KA″ cells.

In converse experiments, activation of Hh signaling by ectopic expression of Shh or dnPKA mRNA induced numerous *tal2*-postive KA″ cells not only along the ventral LFP domain but also in the more dorsal region of the spinal cord (Figure 4A and 4B). Together, these results indicate that Hh signaling is active and required in progenitors of KA″ cells but attenuated and non-essential in differentiated KA″ interneurons.

### Prolonged Hh response interferes with KA″ specification

**Figure 5:**
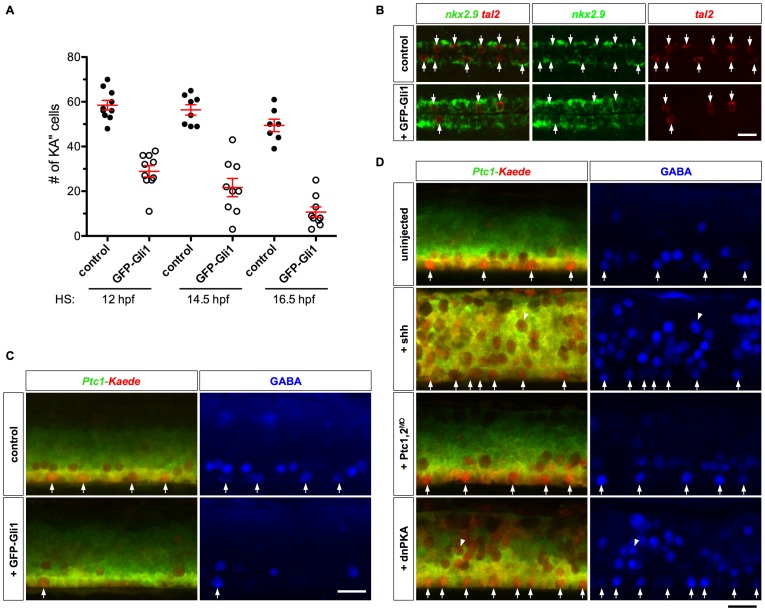
Prolonged Hh response interferes with KA″ specification. (A) Quantification of KA″ specification in embryos overexpressing GFP-Gli1. *hsp-GFP-Gli1* embryos and their non-transgenic sibling controls were heat-shocked at indicated stages, and stained at 25 hpf for the expression of *tal2*. (B) *hsp-GFP-Gli1* and control embryos were heat-shocked at 14 hpf, and stained at 24 hpf for the expression of *tal2* and *nkx2.9*. Induction of GFP-Gli1 results in a reduction of *tal2*-positive KA″ cells (arrows) and expansion of *nkx2.9*-expressing LFPs. (C) *Ptc1-Kaede* control embryos, and *Ptc1-Kaede; hsp-GFP-Gli1* embryos were heat-shocked at 14 hpf, photoconverted at 24 hpf, and stained with the GABA antibody (blue) at 36 hpf. Induced expression of GFP-Gli1 results in a reduction of KA″ cells (arrows). Note that at 36 hpf, GFP-Gli1 expression has minimal contribution to the green fluorescence. (D) *Ptc1-Kaede* control embryos, and *Ptc1-Kaede* embryos injected with Shh mRNA, Ptc1 and Ptc2 morpholinos, or dnPKA mRNA were photoconverted at 24 hpf, and stained with the GABA antibody (blue) at 36 hpf. Arrows indicated GABA-positive cells in the LFP domain. Note that Shh and dnPKA overexpression induced many ectopic GABAergic neurons (arrowheads) throughout the dorsal-ventral axis of the spinal cord, and most of them appeared to lose Hh response by 24 hpf as indicated by the expression of *Ptc1-Kaede^red^*. Scale bars: 20 µm.

The loss of Hh response in KA″ cells raised the question whether termination of Hh signaling is important for cell fate specification. To test this idea, we prolonged Hh response in KA″ cells by expressing a GFP-Gli1 transgene under the control of a heat-shock inducible promoter (*hsp-GFP-Gli1*) [31]. Strikingly, forced expression of GFP-Gli1 reduced the number of KA″ cells with an accompanying increase in *nkx2.9*-expressing LFPs (Figure 5A and 5B). The strongest phenotype was observed when GFP-Gli1 was induced around 16.5 hpf, a stage when most KA″ cells are born but before terminal differentiation markers such as GABA and HuC are expressed (Figure 5A). Since most KA″ cells no longer require active Hh signaling by 16 hpf (Figure 4A), this result suggests that sustained Hh response in differentiating KA″ cells converts them to an LFP-like identity. To test this idea, we induced GFP-Gli1 expression in *Ptc1-Kaede* fish at 14 hpf, and performed photoconversion at 24 hpf. At 36 hpf (12 hours-post-conversion), there was a marked reduction in the number of *Ptc1-Kaede^red^* KA″ cells in the LFP domain (Figure 5C). By contrast, the remainder of the LFP domain expressed *Ptc1-Kaede^red+green^*, indicating continuously active Hh response (Figure 5C). These results suggest that termination of Hh response is essential for KA″ differentiation.

Since forced expression of Gli1 can induce Hh response in differentiating KA″ cells, we asked if activation of upstream components in the Hh pathway can also overcome attenuation of Hh signaling. Injection of Shh mRNA into *Ptc1-Kaede* transgenic fish resulted in substantial up-regulation and expansion of Kaede expression and generated more KA″ cells, but strikingly, KA″ cells expressed little *Ptc1-Kaede^green^* 12 hours after photoconversion at 24 hpf (Figure 5D). This result suggests that even in the presence of ectopic Shh, KA″ cells turn off Hh response before their LFP neighbors. We observed similar results when Hh signaling was activated by depletion of both Ptc1 and Ptc2, or ectopic expression of dnPKA (Figure 5D). In both cases, ectopic KA″ cells were induced but Hh response was terminated. These results reveal that the attenuation of Hh response in KA″ cells is refractory to activating perturbations upstream of Gli transcription factors.

### Notch signaling maintains Hh responsiveness

**Figure 6:**
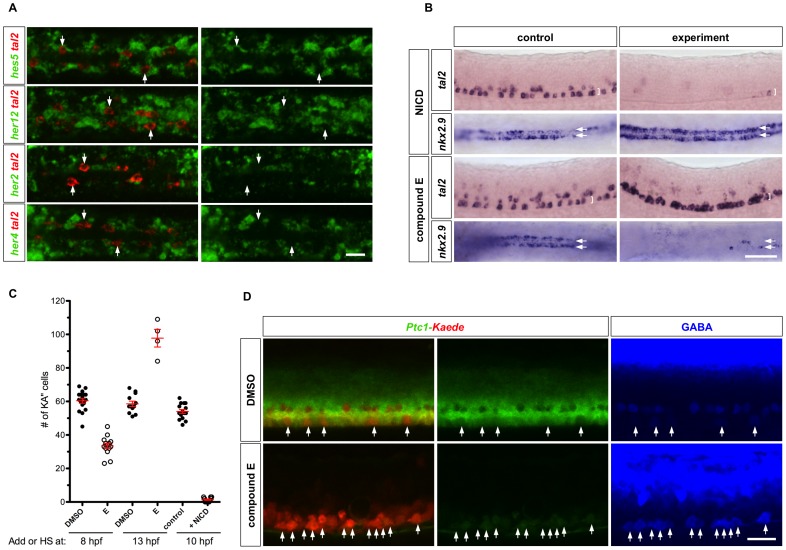
Notch signaling in KA″ specification. (A) Wild type embryos at 18 hpf were double labeled with *tal2* (red) and *hes5*, *her12*, *her2*, or *her4* (green). *tal2*-expressing KA″ cells (arrows) lack expression of *hes5*, *her12*, *her2*, or *her4*. Note that *hes5*, *her12*, *her2*, and *her4* are expressed in only a subset of LFP cells, which might reflect the oscillating nature of these genes in neural progenitor cells as previously reported [49]. Dorsal views are shown. (B) *hsp-Gal4; UAS-NICD* embryos and non-transgenic control embryos were heat-shocked at 10 hpf, and stained at 25 hpf for the expression of *tal2* and *nkx2.9* (top panel). Embryos were treated with DMSO or compound E at 13 hpf, and stained at 25 hpf for the expression of *tal2* and *nkx2.9* (bottom panel). *tal2* and *nkx2.9* staining is shown in lateral and dorsal views, respectively. Brackets indicate the extent of the LFP domain in lateral views, and arrows denote the two rows of LFP domains in dorsal views. (C) Quantification of KA″ specification under different conditions. KA″ cells were scored by the expression of *tal2* at 25 hpf. Note that the fewer data points in compound E treatment at 13 hpf is due to the fact that most compound E-treated embryos had many *tal2*-positive cells in close clusters which prevents reliable scoring. Data shown therefore underestimate the total number of KA″ cells under this condition. (D) *Ptc1-Kaede* embryos were treated with DMSO or compound E at 14 hpf, photoconverted at 25 hpf, and stained with the GABA antibody (blue) at 37 hpf. Compound E treatment induced ectopic GABA-positive cells in the LFP domain (arrows), and abolished the expression of *Ptc1-Kaede^green^* (middle panel). Scale bars: 20 µm in A, D and 50 µm in B.

Notch signaling plays an important role in maintaining neural progenitor cells. We therefore hypothesized that active Notch signaling might not only maintain LFP progenitor state but also maintain Hh responsiveness. To test this model, we first analyzed endogenous Notch signaling activity by examining the expression pattern of known Notch target genes in the LFP domain, including *hes5*, *her12*, *her2*, and *her4* [32]. Strikingly, transcripts of all four genes were largely absent in *tal2*-expressing cells at 18 hpf (Figure 6A). This result suggests that Notch signaling activity is attenuated in KA″ cells.

To test the role of Notch signaling in KA″ formation, we activated Notch signaling by inducing the expression of the constitutively active Notch intracellular domain (NICD) in *hsp-Gal4; UAS-NICD* double transgenic fish [33]. Induction of NICD at 10 hpf almost completely eliminated *tal2*-expressing cells but increased the expression of the LFP marker *nkx2.9* in the LFP domain (Figure 6B and 6C). This result suggests that activation of Notch signaling prevents differentiation of KA″ cells.

In converse experiments, we blocked Notch signaling using compound E, a γ-secretase inhibitor that prevents the generation of NICD [34]. Early blockage of Notch signaling from 8 hpf to 25 hpf resulted in a significant reduction of *tal2*-expressing cells in the LFP domain (Figure 6C). This result is consistent with previous studies that showed that early inhibition of Notch signaling leads to the premature differentiation of neural progenitors into early-born neurons (e.g. primary motor neurons) at the expense of late-born neurons (e.g. KA″ interneurons) [17], [18]. Strikingly, we found that later inhibition of Notch signaling (from 13 hpf until 25 hpf) almost completely transformed the LFP domain into *tal2*-expressing KA″ cells (Figure 6B and 6C). Conversely, expression of the LFP marker *nkx2.9* was mostly absent (Figure 6B). Together, these results suggest that Notch signaling plays an essential role in KA″ induction: downregulation of Notch signaling leads to differentiation of all LFP cells into KA″ cells whereas activation of Notch signaling prevents KA″ differentiation.

We next determined whether Notch signaling plays a role in regulating the timing of Hh response. We performed PHRESH analysis in the presence of Notch signaling inhibitor. We incubated *Ptc1-Kaede* fish with compound E starting at 14 hpf and carried out photoconversion experiments at 24 hpf. At 12 hours-post-conversion, most cells in the LFP domain expressed *Ptc1-Kaede^red^* and co-expressed GABA (Figure 6D). The lack of newly synthesized *Ptc1-Kaede^green^* expression after photoconversion compared to DMSO-treated controls suggests that in the absence of Notch signaling most cells within the LFP domain no longer respond to Hh signaling (Figure 6D). This phenotype was not restricted to the ventral domain, as dramatic reduction of *Ptc1-Kaede^green^* expression was observed throughout the entire spinal cord (Figure 6D). These results suggest that Notch signaling is required for the maintenance of Hh responsiveness.

### Interaction between Hh and Notch signaling in KA″ specification

**Figure 7:**
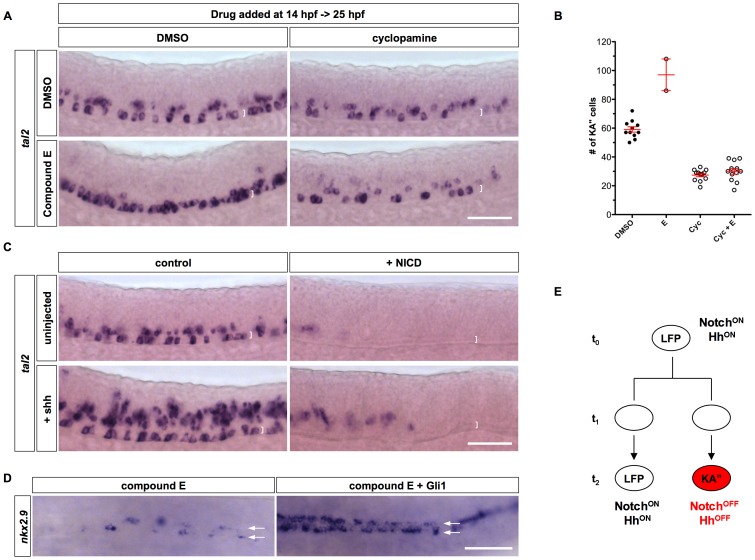
Interaction between Notch and Hh signaling in KA″ specification. (A) Embryos treated with DMSO alone, compound E, cyclopamine, or both compound E and cyclopamine from 14 hpf to 25 hpf, were stained for the expression of *tal2*. (B) Quantification of total number of KA″ cells in experiments shown in A. Note that the fewer data points in compound E treatment is due to the fact that most compound E-treated embryos had many *tal2*-positive cells in close clusters which prevents reliable scoring. The two data points thus underestimate the total number of KA″ cells in E-treated embryos. Cyc: cyclopamine. (C) *hsp-Gal4; UAS-NICD* embryos and non-transgenic sibling controls were left uninjected or injected with Shh mRNA, heat-shocked at 11.5 hpf, and stained at 24 hpf for the expression of *tal2*. Brackets in A and C indicate the dorsal-ventral extent of the LFP domain. Scale bars: 50 µm. (D) *hsp-Gli1* embryos and non-transgenic controls were injected with compound E at 15 hpf, and heat-shocked at 16 hpf, and stained at 22 hpf for the expression of *nkx2.9*. Arrows denote the two rows of LFP domains in dorsal views. (E) Model of KA″ specification. At the early stage (t_0_), LFP progenitors have high level of Notch signaling activity and thereby maintain the progenitor state and Hh responsiveness (Notch^ON^ Hh^ON^). Active Hh signaling in progenitor cells is required for specifying the KA″ identity in subsequent cell divisions. LFP progenitors can undergo three different types of divisions: symmetric LFP/LFP divisions, asymmetric KA″/LFP divisions (shown here), and symmetric KA″/KA″ divisions. Divisions of LFP progenitors at t_1_ generate daughter cells with similar competence to either acquire the KA″ fate or maintain the LFP progenitor fate. Cell-cell interactions or stochastic fluctuations in Notch signaling result in cells with different levels of Notch signaling (t_2_). Cells that maintain high levels of Notch signaling will remain as LFP progenitors and continue to respond Shh (Notch^ON^ Hh^ON^). In contrast, cells that have attenuated Notch signaling will lose Hh response and differentiate into KA″ interneurons (Notch^OFF^ Hh^OFF^). Since sustained Notch or Hh signaling disrupt the differentiation of KA″ cells, formation of KA″ cells initially depends on the activation and then the attenuation of Notch and Hh signaling.

Our results indicate that attenuation of both Notch and Hh signaling is essential for KA″ differentiation. To further clarify the interactions between Notch and Hh signaling in KA″ specification, we first tested whether the induction of ectopic KA″ cells by Notch inhibition depends on active Hh signaling. Inhibition of Notch signaling by compound E from 14 hpf to 25 hpf lead to a more than 50% increase in the number of KA″ cells (Figure 7A and 7B). Concurrent treatment with compound E and cyclopamine at 14 hpf resulted in ∼50% reduction in KA″ cells at 25 hpf similar to cyclopamine treatment alone (Figure 7A and 7B). In contrast, blocking late Hh signaling at 18 hpf with cyclopamine, following compound E treatment at 14 hpf, had no effects on the induction of ectopic KA″ cells (data not shown). These results are consistent with the observation that KA″ cells no longer require Hh signaling after 18 hpf and indicate that the generation of KA″ cells by Notch inhibition depends on early Hh signaling.

In converse experiments, we compared the effects of activating Notch and Hh signaling alone or together. Shh overexpression alone induced ectopic KA″ cells (Figure 4A, 4B and Figure 7C). In contrast, simultaneous activation of both Notch and Hh signaling by NICD and Shh overexpression resulted in very few *tal2*-positive cells in the LFP domain, similar to the NICD-overexpressing phenotype (Figure 7C). These results indicate that the generation of KA″ cells by Hh signaling depends on the inhibition of Notch signaling.

Our results revealed that Notch signaling maintains Hh responsiveness and promotes the LFP over the KA″ fates (Figure 6B and 6D). To establish a causal link between these two functions, we tested whether sustained Hh response is sufficient to bypass the requirement of Notch signaling to induce the LFP fate. Inhibition of Notch signaling by compound E markedly reduced the expression of the LFP marker *nkx2.9* (Figure 6B and Figure 7D). By contrast, ectopic expression of Gli1 induced *nkx2.9* expression even in the presence of compound E (Figure 7D). This result suggests that the primary role of Notch signaling in promoting the LFP over the KA″ fate is mediated through maintaining Hh response.

## Discussion

Using zebrafish lateral floor plate development as a model system, we address how cells switch from progenitor states to differentiated fates. We introduce a novel method (PHRESH) to study signaling dynamics in vivo and provide three major findings. First, sibling cells within the LFP domain in the spinal cord display distinct Hh responsiveness depending on their differentiation state. Second, timely attenuation of Hh signaling is essential for KA″ cell differentiation. Third, Notch signaling maintains LFP progenitor state and Hh responsiveness.

### PHRESH—a novel method to analyze signaling dynamics

Our study presents a novel method, PHRESH, to study the dynamics of Hh signaling in vivo using a *Ptc1-Kaede* reporter. Photoconverted and newly synthesized Kaede protein can serve as a marker to distinguish early and late Hh response, respectively. In conjunction with cell-specific markers, one can determine the temporal profile of Hh response of any cell in any time window during embryogenesis. The Kaede reporter has several advantages over traditional approaches such as GFP reporters, immunohistochemistry or RNA in situ hybridization. First, the Kaede reporter can easily distinguish early and late Hh response in live embryos, whereas perdurance of GFP protein often masks such differences. Second, the Kaede reporter can be photoconverted repeatedly in the same embryo to study the fluctuation of signaling response of the same cells over time. By contrast, immunohistochemistry and RNA in situ hybridization are limited to a single time point in fixed embryos. Finally, Kaede or any other photoswitchable fluorescent protein can be easily adapted to study the dynamics of other signaling pathways and processes.

### Cell lineage and Hh response during spinal cord development

Domains and cell fates within the spinal cord are defined by morphology and gene expression but it is often unclear how neighboring cells are related with respect to lineage and exposure to extrinsic cues. Using in vivo time-lapse imaging, we found that the heterogeneity of the LFP domain results from cell divisions of common progenitors rather than the intermixing between independently generated cell types. Cell divisions within the LFP domain gave rise to three different pairs of daughters: LFP/LFP, KA″/LFP, and KA″/KA″. This combination of symmetric and asymmetric cell divisions might be a general feature of spinal cord development, because previous fate mapping experiments in the motor neuron domain showed that *olig2-GFP* expressing cells can give rise to motor neurons and several types of interneurons [35].

Strikingly, the LFP domain has a “salt-and-pepper” organization: KA″ cells are separated by one to four LFP cells, and two immediately adjacent KA″ cells are extremely rare. How do symmetric and asymmetric divisions contribute to this pattern? Our data suggest that the discontinuous pattern is not generated by distinct modes of cell divisions. First, positions of mother cells along the dorsal-ventral and medial-lateral axes do not correlate with the type of divisions or positions of daughter cells. Preliminary data also suggest that orientation of the division axis does not predict the type of divisions (Huang and Schier, unpublished results). Second, inhibition of Notch signaling converts most LFPs to KA″ cells, suggesting that all LFP cells have the potential to become KA″ cells. Although asymmetric segregation of cell-fate determinants might also contribute to differential Notch signaling activity in daughter cells, our results are consistent with the idea that the three different types of cell divisions generate daughters that are initially equivalent. In this model, daughter cells have similar competence to acquire the KA″ fate or maintain the LFP fate. It is therefore possible that extrinsic cues determine the sites of KA″ differentiation and LFP maintenance.

The shared location of LFP cells and KA″ cells next to the Shh-expressing medial floor plate raised the possibility that they show the same response to Shh. However, we discovered differential Hh response in the LFP domain: all KA″ cells lose Hh response while their neighboring LFP cells continue to respond to Shh. Analogous studies in the mouse spinal cord have shown that expression of *ptc1* is largely absent from the mantle zone, where differentiated neurons reside [36]. These observations suggest that KA″ cells lose Hh responsiveness as they become terminally differentiated and express markers such as GABA and HuC. By contrast, LFP cells remain in a progenitor state: they express progenitor markers such as *nkx2.9* and *nkx2.2a* and continue to incorporate BrdU (data not shown). These results suggest that cells within the same domain in the spinal cord can exhibit distinct temporal profiles of Hh response: progenitor cells remain responsive to Hh while neurons lose Hh responsiveness upon differentiation. Differential Hh responsiveness might be a general feature of spinal cord development, because we also observed loss of Hh response in other types of differentiated neurons, such as motor neurons and KA′ interneurons (Video S2).

### Loss of Hh response is important for cell fate specification

KA″ cells lose Hh response upon differentiation, raising the question how cells regulate Hh responsiveness. Since KA″ cells are in direct contact with Shh-expressing medial floor plate cells, the loss of Hh response is unlikely to be caused by ligand inaccessibility. Moreover, misexpression of Shh does not restore Hh response in KA″ cells. It has been shown that Ptc1 is required for the desensitization and downregulation of Hh signaling over time, suggesting that it might be the mechanism for signal termination [9]. This scenario is also unlikely, however, because depletion of both *ptc1* and *ptc2* or overexpression of dnPKA did not restore Hh response. Repression of *gli2* expression has been implicated in the loss of Hh response in mouse and chick floor plate cells [36]. In support of regulation at the level of Gli transcription factors, induced expression of Gli1 prolongs Hh response. These results suggest that the attenuation of Hh signaling occurs downstream of Shh, Ptc1 and Smo, at the level of Gli transcription factors.

Our results indicate that termination of Hh response is essential for cell fate specification. While early activation of Hh signaling induces KA″ cells, late induction of Gli1 expression prevents attenuation of the Hh response and results in a substantial reduction of KA″ cells. Attenuation of Hh signaling might be a general feature of spinal cord differentiation. For example, induction of the floor plate in chick also depends on transient high-level Hh signaling and subsequent downregulation [36]. Similarly, studies in chick suggest that Hh signaling is lost in postmitotic motor neuron precursors [37].

Premature termination of Hh signaling does not transform LFP cells into KA″ cells. This result suggests that termination of Hh signaling is necessary but not sufficient to induce a specific cell fate. Mechanisms that attenuate responsiveness might ensure that differentiated neurons maintain their fate even upon continuous exposure to inductive signals. In addition, alternative pathways might be activated by the same signals without interfering with cell fate. For example, Gli-independent Hh signaling has been implicated in axon guidance [38]. Thus, blocking Hh responsiveness at the level of Gli transcription factors might allow the activation of a parallel Hh pathway that induces morphological but not transcriptional changes.

### Notch signaling in maintaining neural progenitor cell

Notch signaling has been implicated in maintaining neural progenitors and thereby inhibiting neural differentiation. Our study reveals a novel role for Notch signaling in maintaining the responsiveness of neural progenitor cells to Shh. First, Hh responsiveness of a cell correlates with Notch signaling activity: LFP progenitor cells have active Notch signaling and remain Hh responsive, whereas differentiated KA″ cells lack Notch signaling and lose Hh response. Second, manipulation of Notch signaling activity alters Hh responsiveness and the differentiation program: inhibition of Notch signaling results in loss of Hh response and leads to premature differentiation, whereas constitutive activation of Notch prevents differentiation. Third, sustained Hh signaling through overexpression of Gli1 is sufficient to induce the LFP fate in the absence of Notch signaling. Fourth, Notch signaling plays a permissive rather than an instructive role in KA″ specification. For example, early blockage of Notch signaling at 8 hpf induces ectopic motor neurons, whereas inhibition of Notch signaling at 14 hpf results in ectopic KA″ cells. This result suggests that the role of Notch signaling in cell fate specification is context-dependent. Furthermore, induction of ectopic KA″ cells by repressing Notch signaling requires intact Hh signaling activity, as blockage of Hh and Notch signaling together results in compromised KA″ specification similar to loss of Hh signaling alone.

What is the mechanism by which Notch signaling maintains Hh responsiveness? Our results show the attenuation of Hh signaling occurs at the level of Gli transcription factors. Since activation of Notch signaling by NICD overexpression is not sufficient to induce the expression of *gli* genes in the absence of Hh signaling (Figure S3), regulation of Hh responsiveness by Notch signaling is unlikely to occur at the level of *gli* transcription. Notch signaling might be required to prevent degradation of Gli proteins or their co-factors or prevent the accumulation of a transcriptional repressor of Gli proteins and thereby maintain Hh response. In this scenario, overexpression of Gli1 might titrate out the endogenous signal termination machinery and result in sustained Hh response. Identifying the molecular link between Notch and Hh signaling will be the key in understanding how cells coordinate the transition from the progenitor state to the differentiated state.

In summary, our study suggests the following steps for KA″ specification (Figure 7E): (1) active Notch signaling in progenitor cells maintains Hh responsiveness. (2) Hh signaling induces the identity of progenitors. (3) Loss of Notch signaling leads to loss of Hh response and initiation of KA″ differentiation. Thus, formation of KA″ cells initially depends on the activation and then the attenuation of Notch and Hh signaling.

## Materials and Methods

### Zebrafish strains

Zebrafish strains were maintained and raised under standard conditions. Transgenic fish lines used in this study were: *Gata2-GFP* [27], *Ptc1-Kaede*, *hsp-Gal4* [33], *UAS-NICD* [33], *nkx2.2a-mEGFP* [39], *hsp-GFP-Gli1* [31], and *hsp-Gli1*. To generate *Ptc1-Kaede* transgenic fish, BAC clone zC226H23 from the CHORI-211 library that contains *ptc1* genomic region was selected for bacteria-mediated homologous recombination following standard protocols [40]. A cassette containing the Kaede open reading frame with a SV40 polyA signal and the kanamycin resistant gene was recombined into zC226H23 to replace the first exon of the *ptc1* gene. Successful recombinants were confirmed by PCR analysis. Recombinant *Ptc1-Kaede* BAC was then injected into wild-type embryos, and transgenic lines were established by screening for Kaede expression.

### Timelapse imaging and processing

*Gata2-GFP* embryos were scatter labeled with *H2B-mCherry* by injecting RNA into a single blastomere at 16–32-cell stage. Embryos with mosaic labeling of the neural plate and bright red fluorescence were selected for imaging. Embryos at 3-somite stage (11 hpf) were embedded in 0.7% low melting point agarose and imaged dorsally using either a Zeiss LSM 710 confocal microscope or a Zeiss 2-photon microscope. Z-stacks of the red channel were collected at 2-min intervals for 11 hours, and a high-resolution stack of both the green and red channels was acquired to determine *Gata2-GFP*-positive cells in the lateral floor plate. Additional high-resolution stacks were taken 1 to 2 hours after the final time point to confirm that all KA″ cells turned on the expression of *Gata2-GFP*. Cell divisions were tracked manually with Zen (Zeiss). The original datasets were rendered to make 3D projection images in FluoRender2.8 [41]. The images were compiled to movies with ImageJ.

### Photoconversion of *Ptc1-Kaede* fish

Photoconversion was carried out using the epifluorescence setup on the Zeiss LSM 700 confocal microscope. *Ptc1-Kaede* embryos at the appropriate stage were anesthetized and transferred into 2% methylcellulose. A FITC filter set was first used to identify the region for photoconversion, and the size of the target field can be adjusted by the diameter of diaphragm in the epifluorescence light path. Once the target cells were in focus, a DAPI filter set was switched on for approximately 2 minutes. As photoconversion proceeded the cells would appear pink under the DAPI filter set. The time required for complete green-to-red conversion depends on the expression level of Kaede and the intensity of the light source, but can be easily monitored by switching between DAPI, FITC and TRITC filter sets. After photoconversion, fish were transferred back to fish water and recovered in the dark.

### mRNA and morpholino injections

Synthetic mRNA was generated with the mMessage mMachine Kit (Ambion). Embryos were injected at the one-cell stage with 1–2 nl of mRNA solution to achieve the appropriate concentration: 60 pg *shh* and 120 pg *dnPKA*. To achieve scatter labeling, 0.1 nl of *H2B-mCherry* mRNA was injected at 150 pg/nl into a single blastomere of *Gata2-GFP* embryos at 16–32-cell stage. Morpholino oligonucleotides (Gene Tools, LLC) against *ptc1* (5′-TCTCTGGGATCCGAGGCCATAGTCC-3′) and *ptc2* (5′-AGGAGACATTAACAGCCGAGGCCAT-3′) [42] were injected together into embryos at the one-cell stage each at 0.3 pmol per embryo.

### Heat shock experiments

To induce expression from the heat-shock promoter, fish at appropriate stages were transferred to a 2 ml eppendorf tube in a heat block at 37°C for 30 minutes. After heat shock, fish were transferred back to fish water on a petri dish and recovered at 28.5°C.

### In situ hybridization and immunohistochemistry

Whole-mount in situ hybridization and antibody staining were performed according to standard protocols. Probes were *kaede*, and zebrafish *gli1*, *gli2a*, *gli2b*, *gli3*, *her2*, *her4*, *her12*, *hes5*, *nkx2.2a*, *nkx2.9*, *ptc1*, *shh*, and *tal2* [6], [26], [29], [32], [43]–[46]. To quantify KA″ specification, the total number of *tal2*-expressing cells on both sides of the LFP domain was counted between somite 1 and somite 17 (end of yolk extension) around 24 hpf. For immunohistochemistry, the following antibodies were used: mouse monoclonal antibody to HuC (1∶1000, Invitrogen), rabbit polyclonal antibody to GABA (1∶500, Sigma), and chick polyclonal antibody to GFP (1∶200, Aves). For fluorescent detection of antibody labeling, appropriate Alexa Fluor-conjugated secondary antibodies (1∶500, Molecular Probes) were used. To label nuclei, Draq5 (1∶10,000, Biostatus) was used together with secondary antibodies.

### Drug treatment

Embryos at the appropriate stage were treated with cyclopamine (Toronto Chemical) or compound E (Calbiochem) at a final concentration of 100 µM in 1% DMSO. Control embryos were treated simultaneously with an equal concentration of DMSO. Treated embryos were grown to desired stages for analysis. To increase accessibility, compound E can be injected directly into the yolk of embryos at the desired stage at 9 mM for 3–4 nl.

## Supporting Information

Figure S1 Time-lapse imaging reveals different division patterns in the LFP domain. The top panel corresponds to the time-lapse sequence of the region containing cell 1, cell 2 and their daughter cells in Figure 2C and Video S1. Each frame is a projection of confocal slices containing cells of interest. The green channel was switched on after 6.5 hours. The bottom panel shows the time-lapse sequence of the region containing cell 7 in Figure 2C. Each frame corresponds to a single optical slice containing cells of interest. The green channel was switched on at the last time point. KA″ cells and LFP cells are denoted by arrows and arrowheads, respectively. Cell 1, 2, and 7 undergo KA″/LFP, LFP/LFP, and KA″/KA″ divisions, respectively. The time point of each frame is indicated on top. For the final time point, both the merged image and the image with the green channel alone are shown (red boxes). Scale bars: 10 µm.
(TIF)

Figure S2 LFP cells can be reliably identified based on their locations. (A) *Gata2-GFP* embryos were stained with the *shh* probe (green), the GFP antibody (red) and the Draq5 dye (blue) to label cell nuclei. *Gata2-GFP*-positive KA″ cells (short arrows) and *Gata2-GFP*-negative LFP cells (long arrows) flank the *shh*-expressing medial floor plate cells (arrowheads). (B) *Gata2-GFP* embryos were stained with the *tal2* probe (green), the GFP antibody (red) and the Draq5 dye (blue). All *Gata2-GFP*-positive KA″ cells (short arrows) also express *tal2*. (C) *nkx2.2a-mEGFP* embryos were stained with the *tal2* probe (green), the GFP antibody (red) and the Draq5 dye (blue). All *tal2*-negative cells (long arrows) immediately flanking the medial floor plate (arrowheads) are LFP cells, indicated by the expression of membrane localized EGFP under the control of the *nkx2.2a* promoter (*nkx2.2a-mEGFP*). Dorsal views of embryos at 21 hpf are shown. Scale bars: 20 µm.
(TIF)

Figure S3 Activation of Notch signaling by NICD does not induce the expression of *gli* genes. *hsp-Gal4; UAS-NICD* embryos and non-double-transgenic sibling controls were treated with DMSO or cyclopamine at 4.3 hpf, heat-shocked at 11 hpf, and stained at 24 hpf for the expression of *gli1*, *gli2a*, *gli2b*, and *gli3*. Note that in cyclopamine-treated embryos (right panels), NICD-overexpressing embryos have similar level of expression of *gli* gene as control embryos, indicating that Notch signaling does not induce *gli* transcription in the absence of Hh signaling. Lateral views of embryos are shown. Scale bar: 100 µm.
(TIF)

Video S1 Time-lapse imaging of the LFP domain. The time-lapse movie corresponds to the embryo shown in Figure 2C (left) and Figure S1 (top panels). Cell 1 to 5 and their daughter cells are indicated by arrows. Medial floor plate cells are denoted by white dots. Scale bar: 10 µm.
(AVI)

Video S2 *Ptc1-Kaede* reporter reveals Hh signaling dynamics in vivo. This movie depicts a series of confocal images through the spinal cord of *Ptc1-Kaede* fish that was photoconverted at 25 hpf and imaged at 36 hpf. Motor neurons, KA′ interneurons, and KA″ interneurons (indicated by arrows) express *Ptc1-Kaede^red^*, suggesting that they all terminate Hh response before 25 hpf. Images were taken in lateral views, and each frame corresponds to a 1 µm optical slice. Scale bar: 20 µm.
(AVI)
