# Supplementary figures and images for: Attenuation of Notch and Hedgehog Signaling Is Required for Fate Specification in the Spinal Cord
Source: PLoS Genet. 2012 Jun 7;8(6):e1002762. doi: 10.1371/journal.pgen.1002762 (PMC3369957; doi:10.1371/journal.pgen.1002762)

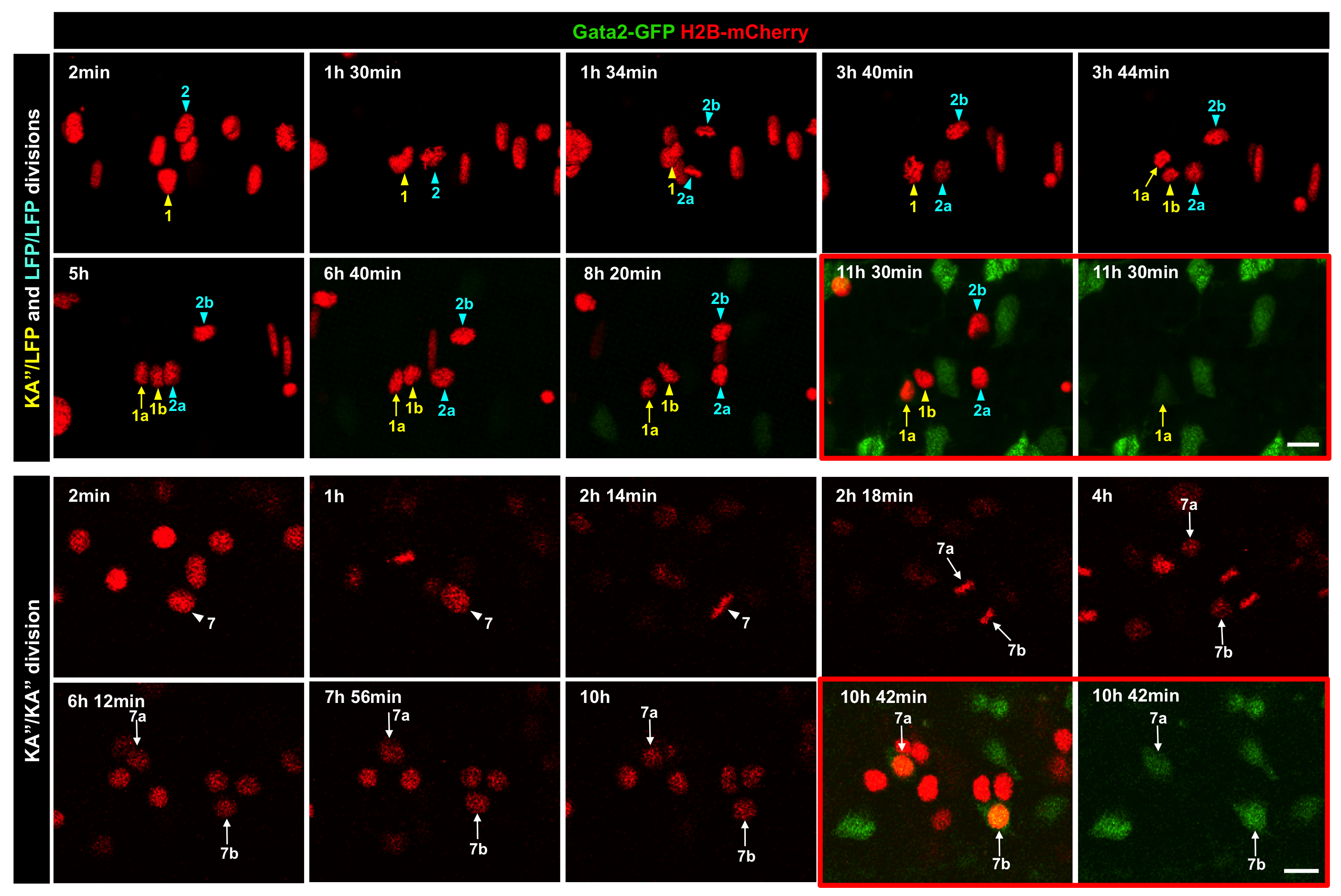

Supplement: Figure S1 — Time-lapse imaging reveals different division patterns in the LFP domain. The top panel corresponds to the time-lapse sequence of the region containing cell 1, cell 2 and their daughter cells in Figure 2C and Video S1. Each frame is a projection of confocal slices containing cells of interest. The green channel was switched on after 6.5 hours. The bottom panel shows the time-lapse sequence of the region containing cell 7 in Figure 2C. Each frame corresponds to a single optical slice containing cells of interest. The green channel was switched on at the last time point. KA″ cells and LFP cells are denoted by arrows and arrowheads, respectively. Cell 1, 2, and 7 undergo KA″/LFP, LFP/LFP, and KA″/KA″ divisions, respectively. The time point of each frame is indicated on top. For the final time point, both the merged image and the image with the green channel alone are shown (red boxes). Scale bars: 10 µm. (TIF) [file pgen.1002762.s001.tif]

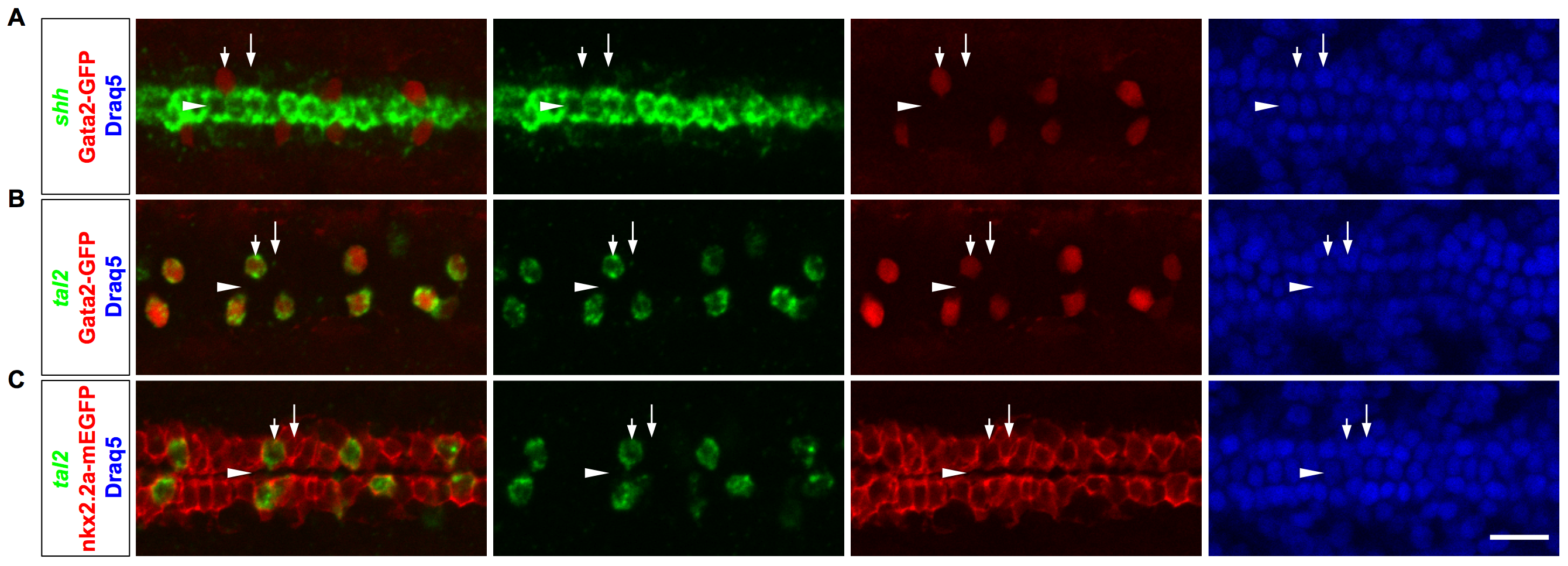

Supplement: Figure S2 — LFP cells can be reliably identified based on their locations. (A) Gata2-GFP embryos were stained with the shh probe (green), the GFP antibody (red) and the Draq5 dye (blue) to label cell nuclei. Gata2-GFP-positive KA″ cells (short arrows) and Gata2-GFP-negative LFP cells (long arrows) flank the shh-expressing medial floor plate cells (arrowheads). (B) Gata2-GFP embryos were stained with the tal2 probe (green), the GFP antibody (red) and the Draq5 dye (blue). All Gata2-GFP-positive KA″ cells (short arrows) also express tal2. (C) nkx2.2a-mEGFP embryos were stained with the tal2 probe (green), the GFP antibody (red) and the Draq5 dye (blue). All tal2-negative cells (long arrows) immediately flanking the medial floor plate (arrowheads) are LFP cells, indicated by the expression of membrane localized EGFP under the control of the nkx2.2a promoter (nkx2.2a-mEGFP). Dorsal views of embryos at 21 hpf are shown. Scale bars: 20 µm. (TIF) [file pgen.1002762.s002.tif]

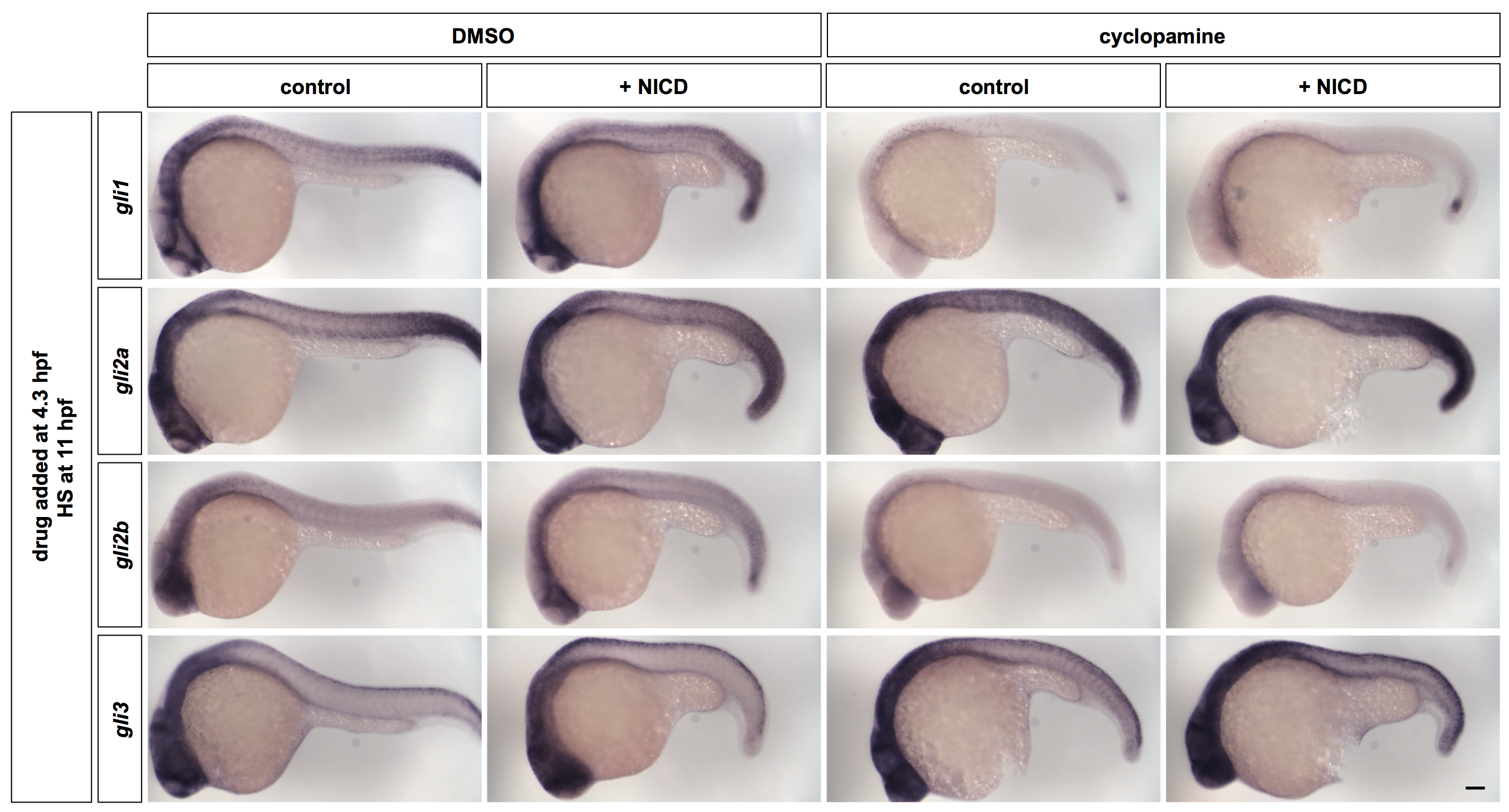

Supplement: Figure S3 — Activation of Notch signaling by NICD does not induce the expression of gli genes. hsp-Gal4; UAS-NICD embryos and non-double-transgenic sibling controls were treated with DMSO or cyclopamine at 4.3 hpf, heat-shocked at 11 hpf, and stained at 24 hpf for the expression of gli1, gli2a, gli2b, and gli3. Note that in cyclopamine-treated embryos (right panels), NICD-overexpressing embryos have similar level of expression of gli gene as control embryos, indicating that Notch signaling does not induce gli transcription in the absence of Hh signaling. Lateral views of embryos are shown. Scale bar: 100 µm. (TIF) [file pgen.1002762.s003.tif]
